# An incidental finding of duodenal gastrointestinal stromal tumor (dGIST): a case report

**DOI:** 10.1097/MS9.0000000000004404

**Published:** 2025-11-25

**Authors:** Absar Nazir, Nadir Khurram Rehman, Fizza Khalid, Arishba Arshad

**Affiliations:** Department of Surgery, Sir Gangaram Hospital, Lahore, Pakistan

**Keywords:** duodenum, gastrointestinal stromal tumor, imatinib, KIT

## Abstract

**Introduction:**

Duodenal gastrointestinal stromal tumors (dGISTs) are rare mesenchymal tumors of the gastrointestinal tract, comprising only 4–5% of them. Usually they are presented with a longstanding history of an abdominal mass, blood in stools or obstruction symptoms. But in our case, the patient presented with a 10-day history of fever along with an abdominal swelling and is recovering well on imatinib therapy after surgery.

**Case Presentation:**

Here, we document a case of an unusual presentation of dGIST with a short history of fever and abdominal mass, which incidentally got diagnosed and treated with surgery and imatinib.

**Clinical Discussion:**

The presentation of dGIST with acute, nonspecific symptoms of fever and abdominal mass is unusual, which delays its diagnosis. Complete surgical resection with or without tyrosine kinase inhibitors remains the mainstay of curative treatment.

**Conclusion:**

This case signifies the importance of considering dGIST even in patients with a short history of abdominal symptoms. Early diagnosis and surgical management helps in achieving favorable outcomes.

## Introduction

The most common mesenchymal tumors of the gastrointestinal tract are the gastrointestinal stromal tumors (GISTs), among which duodenal stromal tumors are only 4–5%^[1,2]^. They are most commonly diagnosed in patients in their sixth decade of life^[3]^. Among duodenal ones, they usually involve the second and third parts of the duodenum^[4]^. Initially being asymptomatic, 5–40% have an incidental diagnosis^[3]^. Then, as their size increases, patients may complain of blood in stools, abdominal pain, abdominal mass, and intestinal obstruction^[4]^.

GISTs tend to arise from interstitial cells of Cajal, involving activating proto-oncogene for tyrosine kinase receptor (KIT) mutations (70%) followed by platelet-derived growth factor receptor alpha (PDGFRA) mutations (10–15%), leading to uncontrolled cellular proliferation^[5,6]^. Though CT is the first investigation sought for the GIST’s diagnosis, and its extent, the final diagnosis is based on histopathology, immunohistochemistry, and tumor marker detection^[6]^.

**HIGHLIGHTS**

- Gastrointestinal stromal tumor (GIST) is an overall rare, but the most common mesenchymal tumor of the Gastrointestinal Tract.
- Duodenal gastrointestinal stromal tumors (dGIST) account for only 4–5% of GISTs.
- dGIST usually presents as an abdominal mass, with symptoms of gastrointestinal bleeding such as melena or hematemesis and may also present with symptoms of obstruction and/or perforation.
- Here, we document a case presented with a short, 10-day history of nonspecific symptoms such as a newly presenting localized abdominal mass with fever which was incidentally diagnosed as dGIST. Therefore, it emphasizes considering dGIST as a differential in such presentations, to achieve optimal outcomes through earlier diagnosis and
- Surgery is the first-line treatment for GIST with imatinib as an adjuvant therapy for intermediate to high-risk cases.

Surgery is the treatment of choice for GISTs, and radical surgical resection (R0) requires exact site, extent, and type of the tumor to be known, though for duodenal gastrointestinal stromal tumors (dGISTs), the vague symptoms and complex anatomy make their diagnosis and surgical resection difficult^[7]^.

Here we present a case of dGIST with an atypical acute presentation, characterized by nonspecific symptoms such as fever and abdominal mass. This case underscores the importance of considering dGIST in the differential diagnosis of such presentations, enabling timely diagnosis and treatment to achieve optimal outcomes.

This case report has been reported in line with the SCARE checklist^[8]^.

## Case presentation

An elderly male in his 70s presented to a tertiary care hospital with the symptoms of fever documented up to 101°F and abdominal swelling for 10 days, which had progressively increased in size along with abdominal pain. There was no history of night sweats, fatigue, loss of weight, blood in stools, or vomiting. On a general physical examination, the patient was febrile and tachycardic, and on focused abdominal examination, a non-tender, pulsatile, well-defined 10 × 8 cm mass was palpated in the left lumbar region. No lymph nodes were palpable. Digital rectal examination was unremarkable. The differential diagnosis of aortic aneurysm (as the mass was transmitting pulsations), any intestinal mass, and abdominal tuberculosis was made.

The patient was managed conservatively on broad-spectrum antibiotics (Meropenem and Linezolid) while he was getting his baseline and specific investigations done.

All baseline investigations, including erythrocyte sedimentation rate (ESR), were within normal limits with only elevated C-reactive protein levels (166 mg/dl) and mild anemia (Hb: 10.2 g/dl).

Ultrasound revealed a well-defined heterogeneous mass with cystic areas and septations measuring 99 × 55 × 85 mm seen above and below the umbilicus, extending towards the left abdomen and left common iliac vessels. No blood flow was seen on Color Doppler. Abdominal CT with contrast showed a retroperitoneal soft tissue density mass 9.25 × 6.64 cm with internal necrosis in the left abdominal cavity and no enhancement (Fig. 1). Normal ESR and CT findings helped in ruling out an aortic aneurysm and abdominal tuberculosis.

**Figure 1.**
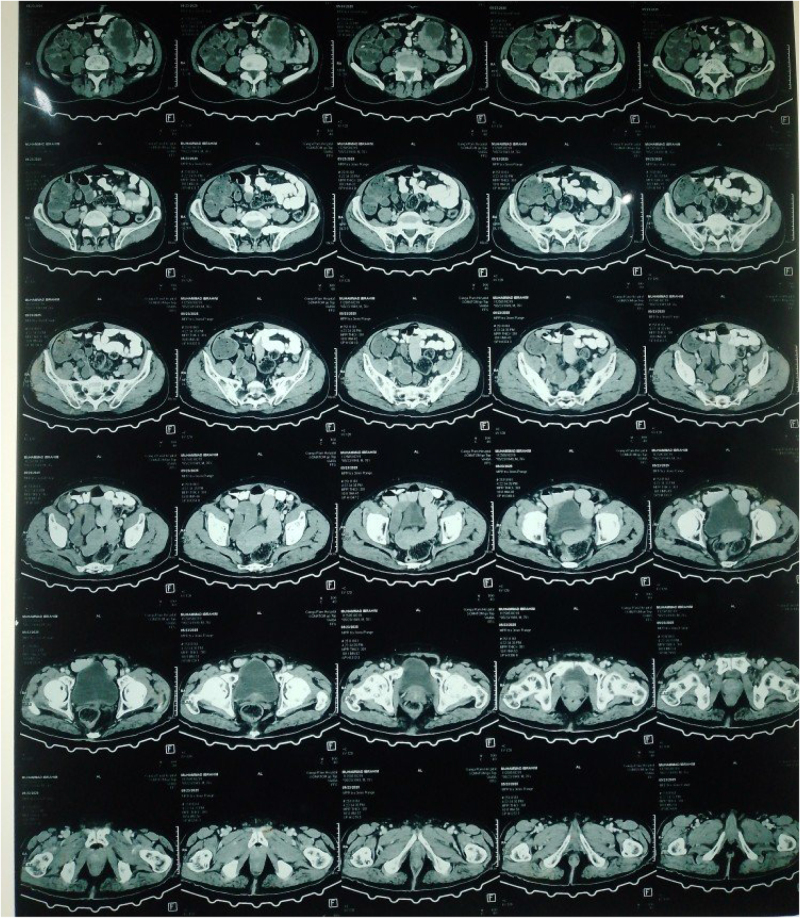
(A) CT abdomen.

**Figure 1.**
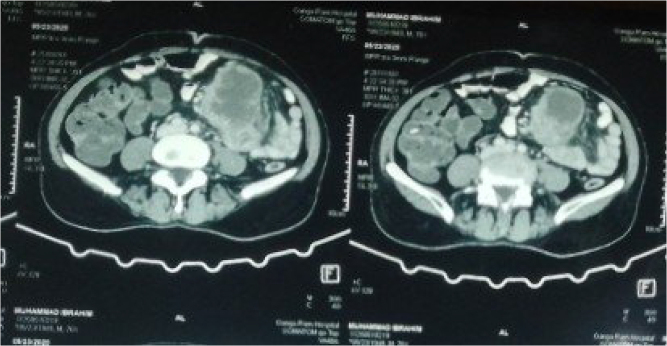
(B) CT abdomen.

Without reaching a final diagnosis, the patient was informed and prepared for an elective exploratory laparotomy after keeping him nil by mouth for 8 h.

**Figure 2.**
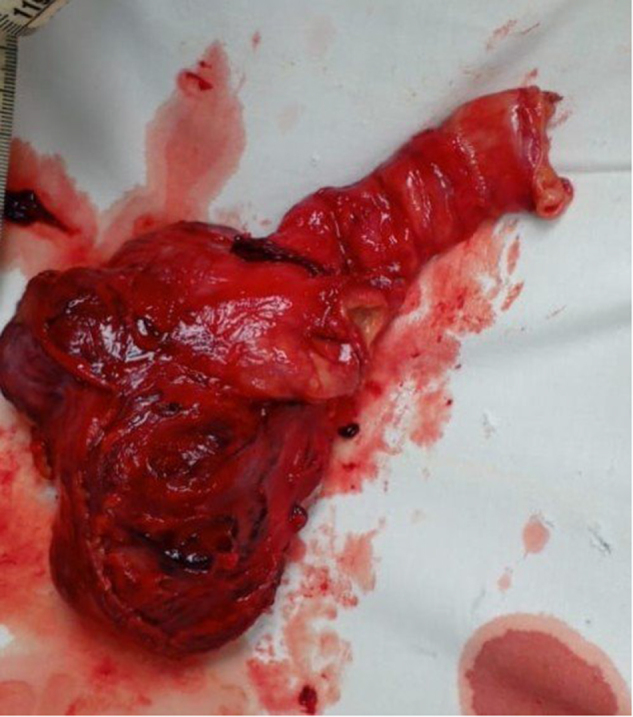
Gross specimen.

Under general anesthesia, initially, a lower midline skin incision was made, considering the location of the mass in the left lumbar region, but it was challenging to remove the mass through this incision and an upward extension of incision was required to remove a 15 × 10 cm cystic to firm mass originating from the third to fourth part of the duodenum with multiple mesenteric adhesions. Upon removal, the appearance of this mass made us consider it GIST (Fig. 2). The mass was excised along with tumor-free margins (5 cm) in the form of a segmental duodenectomy with pancreatic preservation, and a duodenojejunal anastomosis was made. Subhepatic and pelvic drains were placed (which were removed afterward), and the skin was closed with Prolene sutures. A nasogastric tube was passed postoperatively and subsequently removed after 72 h. This challenging surgery was performed by an expert surgeon at our hospital.

**Figure 3.**
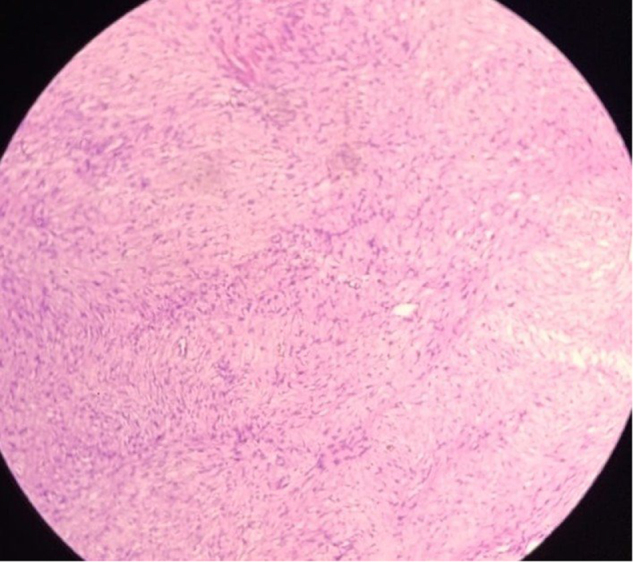
Histopathology.

The specimen was sent for histopathological reporting, which confirmed a high-risk, dGIST of spindle cell type. According to the TNM AJCC staging system^[9]^and Fletcher Classification^[10]^, the patient was put into a high-grade III category as tumor size was 10.5 cm and mitotic rate was 4–5 mitosis/25 high power field (HPF) (Fig. 3 showing a histopathological picture of dGIST of spindle cell type).

Immunohistochemistry was positive for CD117 and DOG1.

Postoperatively, he was given broad spectrum antibiotics and once the patient was stabilized and able to take orally after a few days, he was discharged and imatinib therapy was started due to high risk dGIST, initially on the dose of 100 mg/day (as he was unable to tolerate high doses due to complain of nausea and vomiting) but was gradually increased to 400 mg/day within a period of month. Now he is having regular follow-ups and recovering well.

## Discussion

GISTs account for only 1–3% of GI neoplasms^[1]^, among which duodenal ones are even more rare, comprising only 4–5% of GISTs.

In this case, the patient presented with a short, 10-day history of fever and an abdominal mass in the left lumbar region with no other complaint of blood in vomiting or stools. Although various cases of dGISTs have been reported earlier, they had a typical presentation which helped in their diagnosis. For example, Yadav *et al* reported a case of dGIST presented with black tarry stools and an abdominal mass in the right hypochondriac and right lumbar region^[11].^ Similarly, Hu *et al* documented a case of dGIST presented with a 7-day history of abdominal pain, along with melena and a mass in the right upper abdomen^[6]^.

In our case, there were notable distinctions in terms of the patient’s unusual presenting complaints and location of the abdominal mass, which made dGIST an unlikely initial consideration, thereby delaying its diagnosis. Furthermore, the operation was challenging as the required incision approach was not ideal, and the mass was found to have extensive mesenteric adhesions, adding to the complexity of the operation. This case underscores the importance of including dGIST in the differential diagnosis for such atypical presentations, facilitating earlier diagnosis and timely treatment.

According to Fletcher *et al*, among GISTs, tumor size and mitotic rate are the major prognostic factors indicating risk assessment for recurrence and metastasis^[10]^. Tumors less than 2 cm in size and a lower mitotic rate have a very low risk of recurrence and metastasis and an excellent prognosis, and vice versa for tumors larger than 10 cm^[10,11]^. In our case, the tumor was 10.5 cm in size with a mitotic rate of 4–5 mitosis/25 HPF, putting him in the high-risk category of Fletcher Classification, necessitating imatinib therapy (tyrosine kinase inhibitor targeting KIT) after surgery, which was started postoperatively and was planned to be continued for 3 years.

GISTs were difficult to diagnose and treat previously; however, now, they are diagnosed and differentiated from smooth muscle tumors with the help of immunohistochemistry, KIT, and CD34 positivity. DOG1 is the ideal tumor marker for diagnosing GISTs^[12]^.

Complete surgical resection is the first-line intervention for non-metastasized dGISTs (segmental duodenectomy along with 5 cm tumor-free margins in our case). Various techniques besides extended resection for dGIST, including pancreas-sparing duodenal resection, segmental duodenal resection, and local excision, are used. dGISTs; however, compared to their gastric form, tend to raise greater concern for oncological clearance and tumor-free margins. GISTs <1 cm in size are ideal for limited and wedge resections, given the resultant lumen is wide enough and the Ampulla of Vater can be preserved^[13]^.

GISTs respond poorly to chemotherapy and radiotherapy. With the initiation of imatinib for recurrent, metastatic, unresectable GIST, though the recurrence decreases initially, drug resistance develops in roughly 50% of cases within 2 years^[14]^.

Imaging modalities, including Computerized tomography and magnetic resonance imaging, are the mainstay of follow-up surveillance for GIST, with metastasis being uncommon in them. Frequency depends on the grade of the tumor. Low to moderate grade tumors do not require regular follow-up with an annual CT sufficient for the first 5 years. High-risk tumors require imatinib therapy after surgery for the first 3 years, with CT or MRI every 6 months. More frequent imaging, every 3–4 months, is done for the first 2 years after stopping imatinib therapy, due to the high risk of recurrence. 6–12 monthly imaging is required until 10 years of follow-up. Risk of recurrence after the first 10 years is uncommon^[15]^.

## Conclusion

dGISTs are generally less explored tumors of the GIT, and their resection modalities are not yet clearly defined .They should be studied more and considered in differentials of acute, nonspecific abdominal symptoms, to get better outcomes by earlier diagnosis and management.

## Data Availability

Publicly available.

## Research registration unique identifying number (UIN)

Not applicable as it is a case report. Research registry: None. Unique identifying number or registration ID: None.
